# Restoration Integrity in Primary Teeth Prepared Using Erbium/Yttrium–Aluminum–Garnet Laser: A Randomized Split-Mouth Clinical Study †

**DOI:** 10.3390/children10071215

**Published:** 2023-07-13

**Authors:** Raghdah Abdrabuh, Omar El Meligy, Najat Farsi, Ahmed Samir Bakry, Osama M. Felemban

**Affiliations:** 1 Paediatric Dentistry Department, Faculty of Dentistry, King Abdulaziz University, Jeddah 21589, Saudi Arabia; reabdraboh@kau.edu.sa (R.A.); omeligy@kau.edu.sa (O.E.M.); nfarsi@kau.edu.sa (N.F.); 2 Paediatric Dentistry and Dental Public Health Department, Faculty of Dentistry, Alexandria University, Alexandria 21521, Egypt; 3 Restorative Dentistry Department, Faculty of Dentistry, King Abdulaziz University, Jeddah 21589, Saudi Arabia; drahmedbakry@gmail.com; 4 Department of Conservative Dentistry, Faculty of Dentistry, Alexandria University, Alexandria 21521, Egypt

**Keywords:** Er:YAG laser, restoration integrity, split mouth, Ryge criteria

## Abstract

The most frequently used and universally accepted technique for removing caries is mechanical ablation of decayed tissues by rotating drills. New minimally invasive strategies, such as the use of lasers to perform highly controlled tissue ablation, have been introduced in dental practice. The aim of this study was to assess and compare treatment with a 2940 nm erbium/yttrium–aluminum–garnet (Er:YAG) laser versus a conventional rotary treatment during cavity preparation in children with regard to restoration integrity. In a randomized, controlled, blinded trial using a split-mouth design, 40 (9–12-year-old) children with 80 carious primary molars were included. The cavity in one quadrant was randomized to be treated conventionally using a bur, while the cavity in the other quadrant was prepared using an Er:YAG laser. At the one-year follow-up, clinical examinations were conducted to assess the integrity of the restorations according to the Ryge criteria. The data were analyzed using SPSS version 22 (IBM Inc., Chicago, IL, USA). The average age of the participants was 9.4 ± 1.29 years. Males accounted for 51.4% of the participants. The Ryge criteria showed clinical success of restorations, and there was no discernible difference between the conventional and laser intervention techniques. Over one year, no statistically significant differences in the clinical integrity based on the Ryge criteria were found following class I cavity preparation in primary teeth with either procedure.

## 1. Introduction

The mechanical removal of carious lesions with a rotating handpiece and bur instruments is the most commonly used approach for treating dental caries because it is cost-effective, time-saving, and simple. However, this procedure has many drawbacks, including the potential of removing healthy tooth structures. Recently, laser devices have become an alternative to traditional mechanical rotating instruments for cavity preparation [1].

Dentists use a variety of lasers, the most common of which is the erbium/yttrium–aluminum–garnet (Er:YAG) laser, which was approved by the United States Food and Drug Administration (FDA) in 1997. The Er:YAG laser was approved for use on human teeth and is the most versatile laser in dentistry [2].

The longevity and quality of restorations are important in the success of pediatric dental treatment. Several factors can influence this success, including the method of caries excavation, location of the lesion, the material employed, and operator and patient factors [3]. Few high-quality studies compared restorations produced following caries excavation using laser technology and rotary bur [4]. There have been conflicting reports on microleakage and bonding strength between laser-treated enamel, dentin, and restoration materials [5]. However, studies with a 6-to-24-month follow-up period showed that removing carious tissue using an Er:YAG laser does not negatively affect the clinical success of the restoration [4,6].

Its bactericidal effect on the remaining bacteria in the floor or walls of the cavity, in addition to the absence of any vibration, noise, or strong water jet, render cavity preparation by Er:YAG laser a procedure that is associated with less pain when compared to conventional cavity preparation. In a systematic review, Jacobsen et al. [7] investigated the feasibility of using lasers in caries therapy; the Er:YAG laser was shown to be as effective as rotary burs in eliminating carious tissues. However, due to a lack of high-quality trials, no clear indications about the influence of using lasers on the longevity of restorations or the cost-effectiveness of the method could be formed. Most previous studies conducted on the resin–dentin bond in teeth subjected to different methods of cavity preparation and different types of restoration were in vitro studies. Thus, the purpose of this in vivo study was to compare the integrity of restorations in primary teeth where cavities were prepared with the Er:YAG laser (2490 nm) versus cavities prepared using the conventional rotary method.

## 2. Materials and Methods

### 2.1. Ethical Approval

Ethical approval from the Research Ethics Committee at the Faculty of Dentistry in King Abdulaziz University (KAU) was obtained before starting the project on the 3 April 2019, under approval number 063-02-19. Consent forms were signed by the parents for the participation of their children in the study.

### 2.2. Study Design

A randomized, controlled, blinded split-mouth clinical trial was conducted in the outpatient Department of Pediatric Dentistry at King Abdulaziz University Dental Hospital (KAUDH) in Jeddah, Kingdom of Saudi Arabia, during the time period from 2019 to 2020. The subjects were divided into two groups randomly. The first group included 20 children assigned to receive conventional treatment at the first visit and laser treatment at the second visit (N = 20 children)/(n = 40 teeth). The second group was assigned to receive laser treatment at the first visit and conventional treatment at the second visit (N = 20 children)/(n = 40 teeth). After a follow-up period of one year, an analysis of the restorative integrity was performed using the Ryge criteria.

### 2.3. Randomization

The randomization was performed with a sealed envelope system used to choose which procedure for cavity preparation should be performed first. Each subject was asked to select one of the 40 identical sealed envelopes. The envelope was then opened, the assignment sequence was read and followed, then the envelope was discarded [8].

### 2.4. Sample Size Calculation

In terms of restoration integrity, the results of a previous study [9] comparing the restoration integrity of laser- and bur-prepared cavities were examined; however, their findings showed relatively few failures, making their findings unhelpful. According to the authors’ assumptions, a sample size of 31 pairs will have 80% power to detect a difference in proportions of restoration integrity of 0.2 when the proportion of discordant pairs is expected to be 0.21, and the analysis method was a McNemar’s test of equality of paired proportions with a 5% two-sided significance level.

### 2.5. Eligibility Criteria

The following were the criteria for inclusion in the study:

1. Healthy, cooperative patients.
2. The patients ranged in age from 9 to 12 years.
3. Each patient had at least two active occlusal cavities reaching into the dentine in a primary molar without pulpal involvement (D3 threshold, WHO system).
4. No spontaneous pain.
5. No dental developmental problems.
6. No abscesses, sinuses, or fistulae.

### 2.6. Treatment Procedure

**Figure 1:**
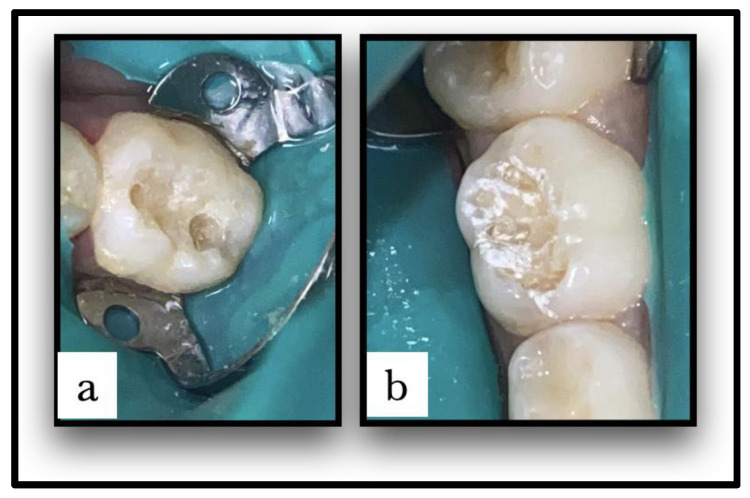
(**a**) Caries removed and cavity prepared with conventional method. (**b**) Caries removed and cavity prepared with Er:YAG laser method.

Caries-affected teeth were randomly assigned to either the conventional caries removal group (group I) or the laser caries removal group (group II); each patient had one carious lesion treated with conventional methods, and the other had caries removal using an Er:YAG laser with a split-mouth design. The cavities were class I cavities, limited to enamel and dentin in the maxillary or mandibular first or second primary molars. The cavities in the conventional caries group were produced using traditional rotating devices, using the MASTER torque high-/low-speed air rotor handpiece (KaVo Dental, Charlotte, NC, USA). The cavities in the laser caries group were created using a non-contact Er:YAG laser handpiece (Doctor Smile, Lambda Scientifica Srl, Vicenza, Italy) with an Er:YAG laser wavelength of 2940 nm. Parameters and the operative mode used for laser hard tissue therapy were 100–200 mJ/20 Hz with water. In both groups, rubber dam isolation and saliva ejector were used. Patients received topical anesthesia before the clamp was placed. Dental caries was excavated until visual inspection showed that the carious lesions had been thoroughly removed (Figure 1). According to the manufacturer’s instructions, cavities were restored using the Clearfil Universal Bond Quick system and Estelite sigma quick, resin-based dental restorative material.

### 2.7. Restoration Integrity Evaluation at the One-Year-Follow-Up Visit

**Figure 2:**
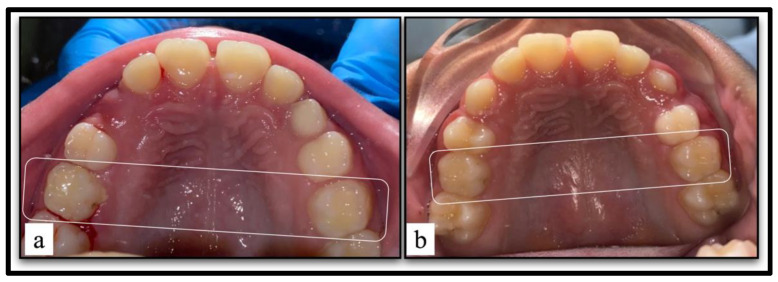
(**a**) The restored teeth at baseline. (**b**) Clinical evaluation of the restorations after one-year follow-up.

The integrity of the restorations was clinically examined one year later at follow-up (Figure 2). The examiner was unaware of the type of treatment received by each tooth [10]. The restorations were evaluated according to Ryge criteria published by the United States Public Health Service [11]. The measured criteria included the following: anatomical form, axial contour, marginal contact, margin discoloration, secondary caries, and visible plaque. Each criterion received a numerical rating from zero to one or two (zero to three for axial contour) which corresponds to scores of Alpha, Bravo, or Charlie (or Delta for axial contour), respectively (Table 1).

### 2.8. Statistical Analysis

The one-year follow-up evaluation consisted of the distribution of Ryge criteria scores in conventional and Er:YAG laser intervention group. To account for the within-subject design, the difference between the intervention groups was examined by McNemar test for each criterion. The significance level was set to *p* < 0.05 (two-tailed test). The data were analyzed using SPSS version 22 ((IBM Inc., Chicago, IL, USA)).

## 3. Results

**Figure 3:**
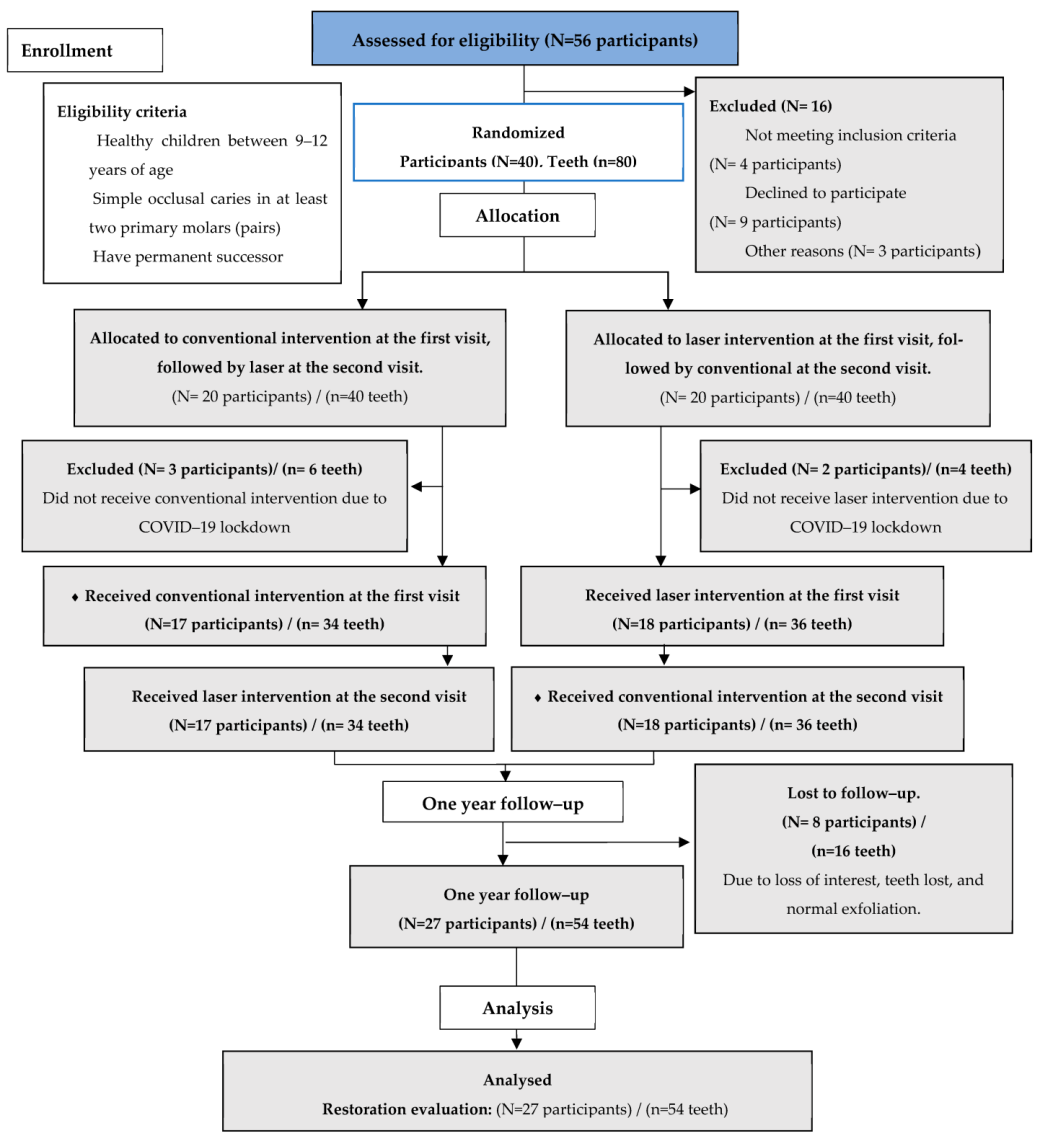
The CONSORT diagram showing patient enrollment. N: number of participants, n: number of teeth.

The first intervention was performed on 40 subjects randomly assigned to the trial; however, 5 could not return for the second session since COVID-19 lockdown was in effect. The restorative assessment analysis was performed in 27 out of 35 participants due to dropouts after one year (N = 27 participants/n = 54 teeth). Of the 54 teeth, 27 were prepared with the conventional method, and 27 with the laser method. A CONSORT diagram showing patient enrollment is presented in Figure 3 [12]. The participants’ (N = 27) mean age was 9.2 ± 1.3 years. There were 15 (55.6%) male and 12 (44.4%) female participants.

### 3.1. Comparison of Ryge’s Criteria between Teeth

The vast majority of restorations were scored “Alpha” in both groups for all of the Ryge criteria (Table 1). When comparing the anatomical form scores’ distribution between restorations, only one (3.7%) of the laser-prepared teeth was rated “Bravo”. For axial contour, three (11.1%) conventionally prepared and one (3.7%) laser-prepared tooth were rated “Bravo”. All restorations were rated “Alpha” when marginal contact was evaluated. For marginal discoloration, “Bravo” was given to two (7.4%) of the teeth that were prepared conventionally and five (18.5%) of the teeth that were prepared using the laser. Three (11.1%) conventionally prepared and three (11.1%) laser-prepared teeth were rated “Bravo” in the secondary caries category. Regarding visible plaque, “Bravo” was given to one (3.7%) conventionally prepared tooth and one (3.7%) laser-prepared tooth.

### 3.2. Comparison of Ryge’s Criteria within Participants

Table 2 compares the quality of the restorations between the two methods considering the within-subject effects inherent in the split-mouth design. The majority of subjects had the same Ryge score on both of their restorations. Since all the restorations with the conventional method were scored as “Alpha” for anatomical form and marginal contact, we could not conduct a statistical test to detect the difference between the intervention groups. The number of subjects with a higher restoration score for the axial contour of conventionally treated teeth was slightly higher (11.1%) than that with restoration scores that were higher in the laser group, but the difference was not statistically significant (*p* = 0.625). The opposite trend was true for marginal discoloration, yet the difference was not statistically significant (*p* = 0.453). Secondary caries and visible plaque also showed no statistically significant difference between the two groups (*p* = 1.00 and *p* = 1.00, respectively).

## 4. Discussion

This study was a randomized, controlled, single-blind clinical trial, allowing within-patient comparisons due to its split-mouth design, with each participant acting as their own control. Split-mouth designs, in which a mouth is divided into two or more experimental portions randomly assigned to various treatments, are commonly employed in clinical research in dentistry. They have a distinct advantage of reducing a significant amount of inter-subject variability from the treatment effect estimate. Many scholars have pointed out that a split-mouth design is only more efficient than a parallel-group design when the within-subject correlation coefficient is significant [13].

The Er:YAG laser technique was used in the present study because of its safe photo-ablation of hard tissue and superior water absorption capacity compared to that of other lasers [14]. After cavity preparation by laser and conventional methods, a total acid etching system with 37% phosphoric acid was used, followed by use of a Clearfil Universal Bond Quick etching primer on the enamel surface. Cavity preparation by laser was followed by acid etching to reduce microleakage at the enamel–composite interface [15,16,17,18,19]. The reason for selecting only one type of adhesive system was to solely obtain a comparison of the cavity preparation techniques [20].

One of the most popularly used criteria for evaluating composite restorations is the Ryge criteria, also known as the US Public Health Service (USPHS) guidelines [21]. These criteria are based on assessing biological, esthetic, and functional parameters and can be adjusted according to the user’s needs [22]. Cvar and Ryge were the first to describe these criteria [12].

In the current study, 16 out of 70 restorations could not be evaluated for integrity due to dropouts after one year. The results of the present study showed that the clinical success of 54 restorations as measured by color matching, marginal integrity, marginal discoloration, anatomic form, and secondary caries was acceptable for the two interventions.

The capacity of the Er:YAG laser to enhance enamel resistance to acid demineralization and decrease acid dissolution may account for the positive result in the current investigation. In addition, the bactericidal impact of the laser system on dentin surfaces has been established by numerous authors [23,24,25,26]. As a result, secondary caries would be avoided [27]. Melting and recrystallization with pores are physical changes also caused by the laser, resulting in a coarse surface that provides a micromechanical bond for adhesives [28].

Another reason for our positive results could be the promotion of acid demineralization resistance by Er:YAG laser irradiation. Several investigations have identified secondary caries as the leading cause of restorative failure. It has been shown that Er:YAG laser irradiation promotes resistance to acid demineralization, thereby lowering the acid dissolution of tooth hard tissues and playing a key role in secondary caries prevention [29].

The present findings are consistent with those of a previous study by Gutknecht et al., who investigated and found that the combination of Er:YAG laser preparation with acid etching showed no difference in the microleakage of composite restorations prepared using laser or bur in class II cavities [30]. The current findings are also consistent with those of the study by Yazici et al., who investigated the effect of bur and laser on class I occlusal resin composite restorations over two years. The modified Cvar/Ryge criteria discovered no significant differences between the two cavity preparation approaches [9]. In a heuristic view, it may be inferred that using an Er:YAG laser to prepare class I cavities had no clinical relevance to the success of composite restorations; however, it may have a beneficial influence on their retention by improving the adhesion of the composite resin to the dental hard tissue.

Long-term clinical trials are required to confirm the last assumption. This study obtained satisfactory results over one year with the Er:YAG laser and a conventional method. In comparative research, Dostalova et al. concluded that while the filling material (composite resin) in laser cavities was relatively durable, cavo-surface margin discolorations of 82–86% with an “Alpha” rating could be an issue, and color and anatomic shape changes were seen in 4–8% of the teeth [31]. Our clinical observations for restorations filled following laser preparation contradict these findings.

## 5. Conclusions

Over one year, no statistically significant differences in the clinical integrity according to the Ryge criteria were produced, following the preparation of class I cavities in primary teeth with either the conventional or the laser caries removal. The Er:YAG laser did not induce any negative effects on the clinical outcome of the restorations when compared to their corresponding restorations prepared with the conventional method.

## 6. Clinical Significance

The results of the present study showed that the clinical success of 54 restorations as measured by color matching, marginal integrity, marginal discoloration, anatomic form, and secondary caries was acceptable for the two interventions.

## Figures and Tables

**Table 1:** Distribution of Ryge criteria scores between teeth (n = 54 teeth).

Criterion/Score			Conventional Method n (%)	Er:YAG Laser Method n (%)
Anatomical form				
0	Alpha	Restoration is continuous with existing anatomical form.	27 (100)	26 (96.3)
1	Bravo	Restoration is not continuous with existing anatomical form, but the dentin and base are not exposed.	0	1 (3.7)
2	Charlie	The lack of continuity is severe enough that the dentin or base is exposed.	0	0
Axial contour				
0	Alpha	Restoration is continuous with existing anatomical form or is slightly flattened or over contoured.	24 (88.9)	26 (96.3)
1	Bravo	A tangentially placed explorer cannot touch two opposing Cavo surface line angles simultaneously.	3 (11.1)	1 (3.7)
2	Charlie	The surface is noticeably concave upon visual inspection. A tangentially placed explorer cannot touch two opposing Cavo surface line angles simultaneously.	0	0
3	Delta	The surface is noticeably concave upon visual inspection, and the base or dentin is exposed. It is under-contoured; hence, the tissue trauma is evident.	0	0
Marginal contact				
0	Alpha	No crevice is visible, and the explorer can be drawn across the surface without catching.	27 (100)	27 (100)
1	Bravo	The crevice is big enough to be visible and for the explorer to catch; however, the dentin and base are not exposed.	0	0
2	Charlie	The crevice is so big that the explorer catches and the dentin or base is exposed.	0	0
Marginal discoloration				
0	Alpha	The color of the margin matches the color of the restoration and/or the adjacent tooth structure.	25 (92.6)	22 (81.5)
1	Bravo	Discoloration is only present at the junction between the restoration and the tooth; however, it has not continued in the direction of the pulp.	2 (7.4)	5 (18.5)
2	Charlie	Discoloration begins at the junction between the restoration and the tooth and continues in a pulpal direction.	0	0
Secondary caries				
0	Alpha	No secondary caries along margin.	24 (88.9)	24 (88.9)
1	Bravo	Secondary caries along margin evident upon visual inspection.	3 (11.1)	3 (11.1)
Visible plaque				
0	Alpha	No visible dental biofilm.	26 (96.3)	26 (96.3)
1	Bravo	Dental biofilm visible due to restorations.	1 (3.7)	1 (3.7)

**Table 2:** Comparison of Ryge criteria within participants (N = 27 participants).

Ryge Criteria	Number of Subjects Where the Restoration of Both Teeth Had the Same Score	Number of Subjects Where the Restoration of the Conventional-Treated Tooth Had Higher (Worse) Score	Number of Subjects Where the Restoration of the Er:YAG Laser-Treated Tooth Had Higher (Worse) Score	*p*-Value
Anatomical form	26 (96.3)	0	1 (3.7)	NA
Axial contour	23 (85.2)	3 (11.1)	1 (3.7)	0.625
Marginal contact	27 (100)	0	0	NA
Marginal discoloration	20 (74.1)	2 (7.4)	5 (18.5)	0.453
Secondary caries	23 (85.2)	2 (7.4)	2 (7.4)	1.00
Visible plaque	27 (100)	0	0	1.00

McNemar test. NA: not applicable because *p*-value could not be calculated since all restorations (100%) in the conventional method group were scored as “Alpha”.

## Data Availability

The data supporting the conclusions of this study can be obtained upon request to the corresponding author at omfelemban@kau.edu.sa.
